# Laparoscopic Resection of a Non-functional, Extra-adrenal Paraganglioma: A Case Report and Literature Review

**DOI:** 10.7759/cureus.7753

**Published:** 2020-04-20

**Authors:** Antonios Katsimantas, Spyridon Paparidis, Dimitrios Filippou, Konstantinos Bouropoulos, Nikolaos Ferakis

**Affiliations:** 1 Urology, Mediterraneo Hospital, Glyfada, GRC; 2 Urology, Korgialenio-Benakio Hellenic Red Cross Hospital, Athens, GRC; 3 Anatomy and Surgical Anatomy, National and Kapodistrian University of Athens Medical School, Athens, GRC; 4 Surgery, National and Kapodistrian University of Athens Medical School, Athens, GRC

**Keywords:** extra-adrenal paraganglioma, laparoscopy, retroperitoneal tumor

## Abstract

Non-functional, extra-adrenal, retroperitoneal paraganglioma is a rare, neuroendocrine, and potentially malignant tumor. Its diagnosis and treatment may be challenging. A 69-year-old female patient was admitted because of a left para-aortic, solid, 4.4-cm mass, incidentally discovered during abdominal ultrasonography for screening purposes. Her clinical examination was unremarkable. Preoperative differential diagnosis based on cross-sectional imaging included tumor of neuroendocrine or mesenchymal origin. Hormonal investigation with 24-hour urinary catecholamines and metanephrines and plasma-fractionated metanephrines was in the normal range. Following consultation with the endocrinologist and anesthesiologist, the tumor was removed by using the three-dimensional (3D) laparoscopic transperitoneal surgical approach. The perioperative course was uneventful and the patient was discharged on the third postoperative day. Histopathologic findings were consistent with the diagnosis of retroperitoneal extra-adrenal paraganglioma of 5 cm in maximum diameter.

## Introduction

Paragangliomas are rare neuroendocrine tumors originating from chromaffin cells along the sympathetic and parasympathetic chains [1]. Parasympathetic paragangliomas are usually inactive and located mostly in the neck and skull base, while sympathetic paragangliomas are mainly located in the retroperitoneum and usually produce and secrete norepinephrine [2,3]. Up to 15% of retroperitoneal paragangliomas are non-functional, and up to 10% are functional without clinical manifestation, although they resemble functional ones histologically and immunologically [4-6]. Their clinical presentation varies depending on their location, size, and secretion status, while their diagnosis and treatment may be challenging due to their close proximity to major vessels and surrounding organs, demanding multidisciplinary medical approach and cooperation [2,7,8]. Our study aims to underline the importance of a multidisciplinary medical approach and the benefit of a minimally invasive surgical method for treating retroperitoneal paraganglioma. We also present a short review of the relevant literature in English.

## Case presentation

In June 2019, a 69-year-old female patient was admitted to the Department of Urology of Korgialenio-Benakio Hellenic Red Cross Hospital, Athens, Greece, due to a tumor within the left para-aortic space. Her medical history included depression. She was given citalopram hydrobromide 10 mg per os on a daily basis. In addition, she was placed under endocrinologist’s surveillance due to the presence of thyroid nodules. Clinical examination was unremarkable.

**Figure 1:**
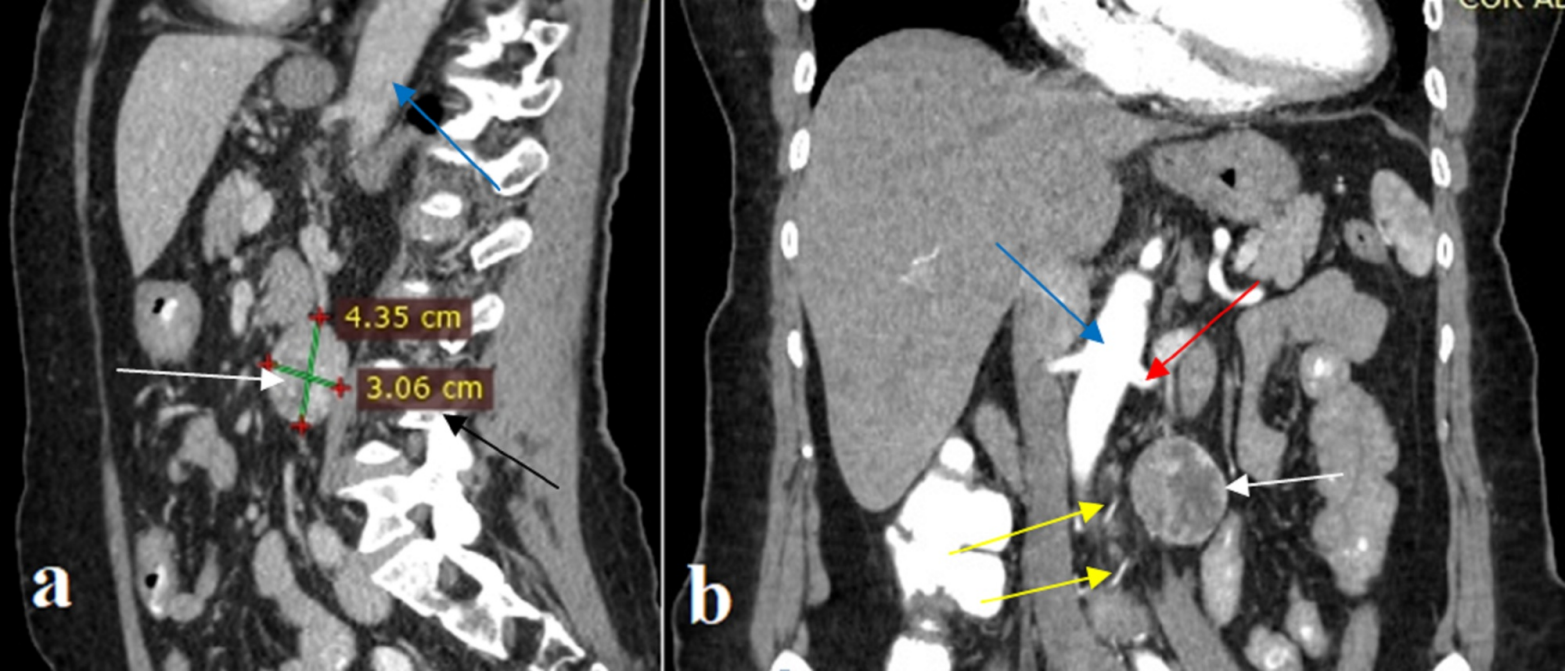
Preoperative abdominal CT scan a: sagittal view; b: coronal view. The images demonstrate a well-defined, left para-aortic, heterogeneous sizable mass (white arrow) located at the height of the fourth lumbar vertebra (black arrow), below the left renal artery (red arrow). Two arterial branches (yellow arrows) of the tumor’s vascular pedicle originating from the aorta (blue arrow) are recognized CT: computed tomography

**Figure 2:**
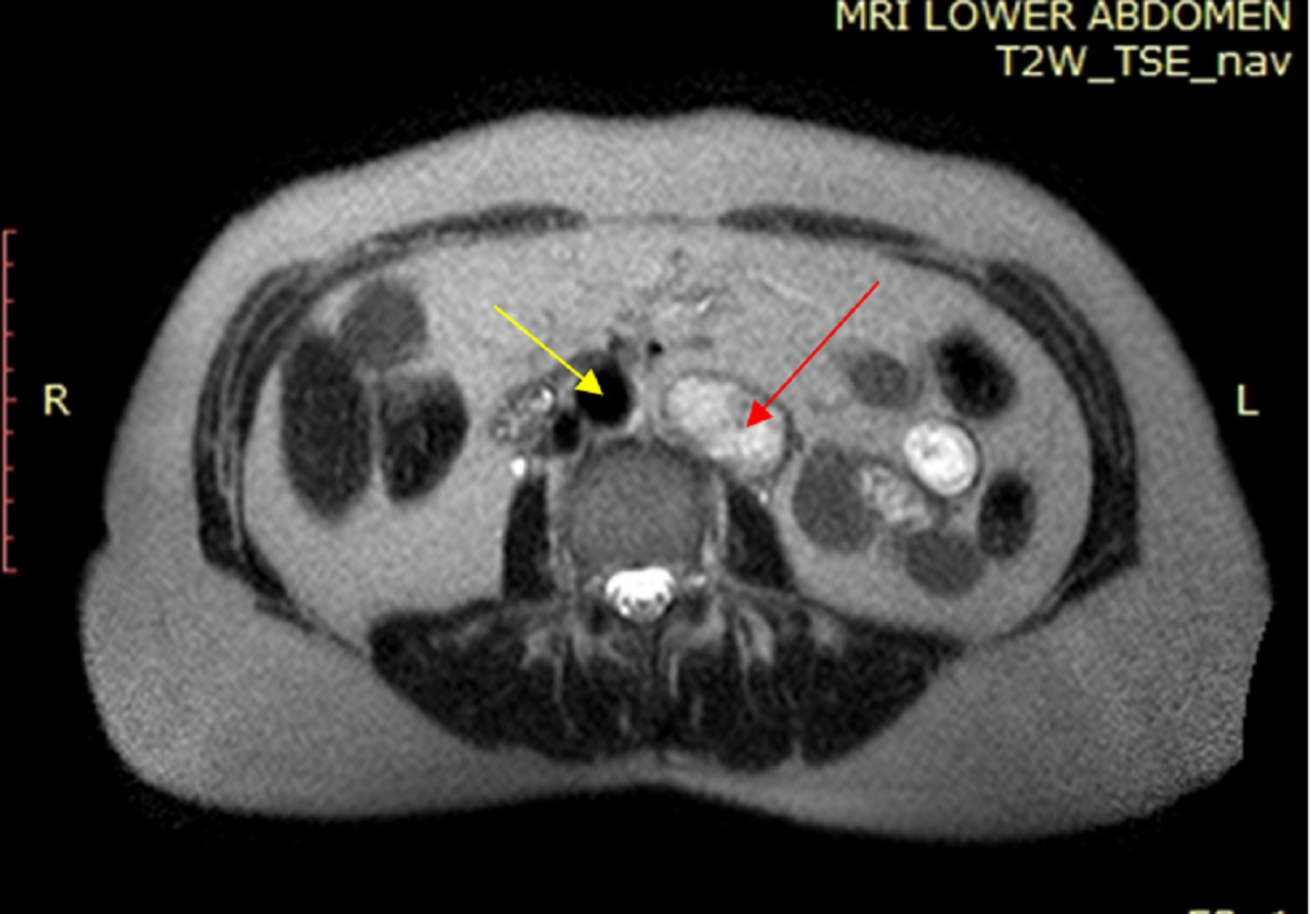
Preoperative T2-weighted axial MRI of the abdomen The image shows the lesion (red arrow) demonstrating a heterogeneous hyperintense signal, located on the left side of the aorta (yellow arrow) MRI: magnetic resonance imaging

The mass had been incidentally discovered during abdominal ultrasonography for screening purposes three months ago. Further investigation with an abdominal CT scan demonstrated the presence of a well-defined, left para-aortic, solid, 4.4-cm mass, located at the height of the fourth lumbar vertebra. The lesion presented heterogeneous enhancement following intravenous administration of contrast agent. The CT scan could not differentiate between the mesenteric mesenchymal lesion and the retroperitoneal tumor (Figure 1). Preoperative MRI confirmed the presence of a solid, well-defined oval tumor 4 x 2.6 x 4.5 cm in size. The mass demonstrated hypointense signal on T1-weighted sequences (similar to skeletal muscles) and heterogeneous hyperintense signal on T2-weighted sequences, without changing its signal on T1 out-of-phase sequences (Figure 2). The mass presented enhancement from periphery to the center following the intravenous administration of paramagnetic contrast agent. The lesion was located in the left para-aortic space and retroperitoneally, inferiorly to the left kidney, and in contact with the inferior surface of the horizontal part of the duodenum, without infiltrating surrounding tissues. Based on preoperative imaging, the differential diagnosis included retroperitoneal tumor of neuroendocrine or mesenchymal origin. Preoperative 24-hour values of urinary catecholamines and metanephrines and plasma-fractionated metanephrines were unremarkable; so was the preoperative cardiological evaluation.

**Figure 3:**
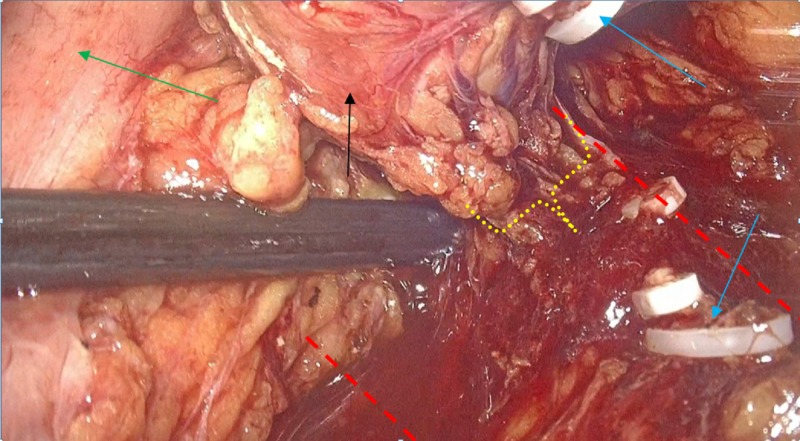
Intraoperative photo The intraoperative photo during the gradual dissection and ligation of the tumor’s vascular pedicle (yellow dot-line bracket). We separate the tumor (black arrow), a ligated and cut arterial branch (blue arrows) originating from the aorta (between red dotted lines), and the descending colon (green arrow) is deflected cephalad-medially by the laparoscopic suction

After obtaining informed written consent, the patient underwent surgical removal of the mass under general anesthesia, by using three-dimensional (3D) laparoscopic transperitoneal, 4-trocars lateral approach with the lesion side up and the table half-flexed. A 3D high-definition camera and a 30° laparoscope were used, while pneumoperitoneum was performed by using an open Hasson technique and was steadily maintained at 12 mmHg. Following the tumor’s exposure, the mass was manipulated gently and the dissection was performed by using monopolar hook diathermy and a harmonic scalpel (Figure 3). No hemodynamic fluctuations were observed during pneumoperitoneum and tumor’s handling. The operative time was 142 minutes. A- or β-adrenergic antagonist had not been administered preoperatively, based on endocrinologist/anaesthesiologist consultation, as the patient had stable blood pressure and heart rate and the diagnosis was still unclear. The patient had an uneventful postoperative course and was discharged on the third postoperative day.

**Figure 4:**
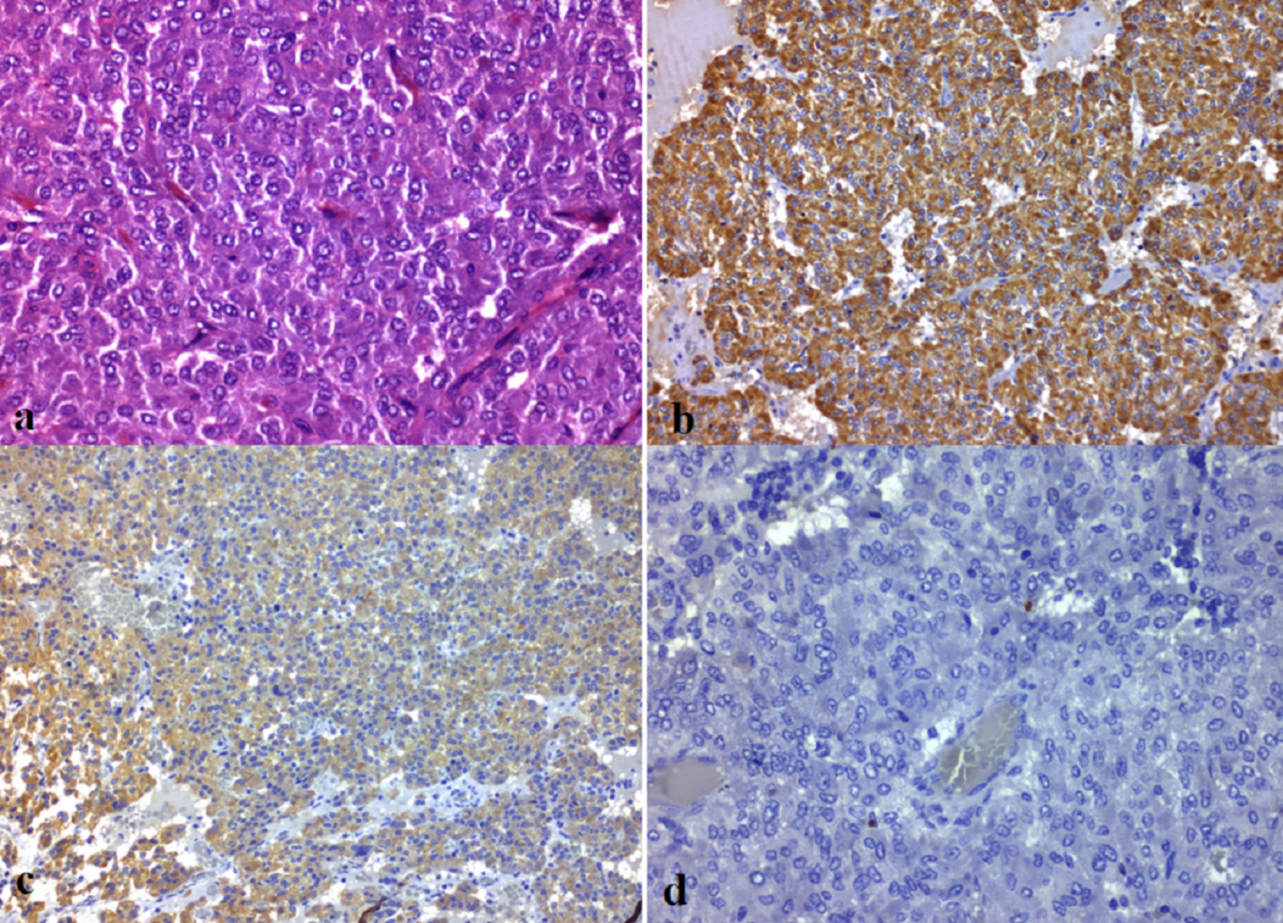
Histological images a: paraganglioma with nested architecture and distinct zellballen pattern separated by fibrovascular stroma (hematoxylin and eosin stain, x400); b: positive immunohistochemical reaction for chromogranin (x200); c: positive immunohistochemical reaction for synaptophysin (x200); d: Ki67 proliferative activity was low (1-2%) (x400)

Macroscopic examination revealed a solid, encapsulated oval 5 cm in size. Microscopically, the mitotic activity was low and the tumor was highly vascular. Tumor cells were mainly polygonal and presented a nested-like growth pattern within a prominent fibrovascular network. They were positive for chromogranin-A (diffusely) and for synaptophysin (focally) and negative for epithelial AE1/3 cytokeratin. Sustentacular cells stained with S100 protein were identified at the periphery of the nests. Histopathologic findings were consistent with the diagnosis of paraganglioma. The cellular proliferation marker Ki67 was low (1-2%) and the tumor’s score was 5 according to the Grading System for Adrenal Pheochromocytoma and Paraganglioma (GAPP), corresponding to paraganglioma of intermediate differentiation (Figure 4). Tumor’s capsule was in contact with the inked surgical resection margin focally, and a suspicious invasion of a microvessel in the area of the capsule was observed.

Three months postoperatively, the patient underwent thyroidectomy, and histology revealed papillary thyroid cancer. Postoperative genetic screening was negative. ^123^I-metaiodobenzylguanidine (MIBG) scintigraphy, abdominal CT scan, and hormonal investigation were negative six months postoperatively, and a rigorous follow-up scheme including outpatient consultations with oncologist and endocrinologist was subsequently scheduled.

## Discussion

Retroperitoneal extra-adrenal paragangliomas are rare tumors originating from chromaffin cells of the primitive neural crest during their migration to form the paraspinal sympathetic paraganglia. They have an estimated incidence of 2-8 per million people in the general population [2,4,7-9]. There is a great variation regarding their location, but 85% of them are retroperitoneal and are usually located between the origin of inferior mesenteric artery and the aortic bifurcation [7,8].

They usually present as a single unilateral tumor; however, in 10-50% of cases, they are part of hereditary syndromes like multiple endocrine neoplasia (MEN) types 2A and 2B, Von Hippel Lindau disease, neurofibromatosis type 1, Carney-Stratakis dyad, and familial paraganglioma associated with mutations in genes encoding succinate dehydrogenases B, C, and D [2,3,10]. The median age at diagnosis in sporadic form is between 20 and 50 years, while the hereditary form is associated with an earlier diagnosis of about a decade [3]. The female-to-male ratio is 3:1 for sporadic forms and 1:1 for hereditary ones [2]. Our patient presented with thyroid cancer, leading us to think that there may be a relationship with the diagnosis of paraganglioma; however, histology revealed papillary subtype, instead of medullary one, which is a feature of MEN type 2 syndrome [11]. Apart from this, our patient presented no features of an associated genetic syndrome, and genetic testing was negative.

Clinical presentation and findings contribute to the diagnosis, although there are asymptomatic forms [3]. The triad of hypertension (persistent or paroxysmal), palpitations, and excessive generalized sweating is the most classical clinical presentation of paraganglioma [2]. Moreover, uncontrolled secretion of catecholamines, which can also be affected by various stresses, may evoke symptoms like dry mouth, facial flushing, dilated pupils, constipation, headache, tremors, and generalized weakness [2,7]. Diabetes and other metabolic disturbances are not uncommon [10]. Late and/or inappropriate diagnosis and treatment may be even life-threatening [10]. Larger tumors may cause symptoms related to compression of surrounding tissues [8]. Non-functional paragangliomas may remain asymptomatic, even in the presence of a sizable mass, as observed in our case [8].

Both preoperative biochemical investigation and imaging modalities play a vital role in the diagnosis of paraganglioma and in subsequent treatment planning [7]. Elevated values of 24-hour urinary catecholamines and metanephrines and plasma-fractionated metanephrines are detected in the case of a functional tumor [2,3]. False-positive test results may be noticed in stressful clinical circumstances (i.e., acute illness) and in case of certain medication intake such as serotonin reuptake inhibitors, norepinephrine reuptake inhibitors, tricyclic antidepressants, levodopa, and antipsychotics [2,11]. Our patient was treated with citalopram hydrobromide, which is an antidepressant of the selective serotonin reuptake inhibitor class, but we did not observe any false-positive results.

CT scanning and MRI are used in order to locate the tumor [3]. MRI is considered the gold standard for the definitive diagnosis [7]. The usual appearance is that of an oval/rounded mass, with a density similar to the liver and muscles [5,7]. The lesion typically has a hypointense signal on T1-weighted sequences and hyperintense signal on T2-weighted sequences, while their hyper-vascularization is responsible for the intense enhancement following intravenous administration of contrast medium [5]. These findings were confirmed in our case; however, we did not notice any necrotic or cystic appearance at the tumor’s center or any calcifications, which may be observed in some cases [5]. Functional tests like ^123^I MIBG scintigraphy, ^68^Ga­-labeled 1,4,7,10-­tetraazacyclododecane-1,4,7,10-­tetraacetic acid-octreotate (DOTATATE) positron emission tomography (PET)/CT, or ^18^F-­labeled L-­dihydroxyphenylalanine (L-DOPA) PET/CT provide a whole-body investigation and are able to differentiate between functional and non-functional paraganglioma and to detect synchronous tumors or metastases, especially if CT scan and MRI are negative and if there is a strong suspicion of catecholamine secreting tumor [3,11,12]. Diagnosis of non-functional paragangliomas is challenging, and they may resemble other epithelial or mesenchymal lesions due to the absence of unique imaging characteristics on cross-sectional imaging, as observed in our case [13]. It is worth mentioning that the widespread use of imaging modalities and the genetic investigation of the family members of patients with hereditary syndromes have resulted in an increase in the incidental detection of tumors in asymptomatic patients in the last few years [11].

Following diagnosis, an α-blocker is administered at a dose progressively up-titrated for at least one to two weeks preoperatively, accompanied by high sodium and fluid intake [10,11]. The aim is to control blood pressure, avoid major intraoperative cardiovascular complications, especially during tumor’s handling and following intrabdominal pressure rise due to the insufflation of carbon dioxide, which may induce catecholamines’ excretion, and prevent hypotensive episodes following tumor’s removal [3,5,10,11,14]. B-blocker may be required in order to control reflex tachycardia following α-adrenergic blockade [11]. Interestingly, Heinze et al. recently reported a case of an undiagnosed retroperitoneal paraganglioma that was complicated by cardiac rhythm abnormalities, hypertensive crisis, and finally by cardiac arrest intraoperatively during laparoscopic excision [1]. The patient finally survived, and this case report underlines the role of an experienced anaesthesiologic team in such circumstances [1,3]. In our case, the differential diagnosis included retroperitoneal paraganglioma, and we performed the required hormonal investigation, which was negative. So, we proceeded to undertake surgical resection without administration of pharmacological preparation following consultations with the endocrinologist and anesthesiologist, and the patient had an uneventful perioperative course, although there are reports of silent retroperitoneal paragangliomas that were operated without α-adrenergic blockade and presented transient rise of blood pressure [5]. In any case, careful perioperative medical approach and cooperation among surgeons, radiologists, endocrinologists, anesthesiologists, and cardiologists are of paramount importance in order to achieve optimum outcomes for the patient [3].

Surgical resection represents the standard of care and can be performed traditionally by open surgery, while there has been a trend towards minimally invasive procedures in recent years [3,4]. Furthermore, as in our case, exact preoperative diagnosis may be impossible to be established and surgical excision of a suspicious mass may be needed [6]. Benefits of laparoscopy include better visualization and accuracy in dissection due to the optical magnification, reduced postoperative pain, earlier bowel mobilization, shorter length of hospitalization, and better cosmesis [3,15]. However, laparoscopic excision of abdominal paraganglioma is a technically demanding procedure, which requires adequate laparoscopic skills [3,15]. Indeed, our experience in laparoscopic surgery of retroperitoneal area resulted in the complete surgical removal of the mass, absence of complications, and short hospitalization time, although this was the first case of retroperitoneal paraganglioma performed by our team. Previous studies have demonstrated that an abdominal paraganglioma may invade major vessels and, in such cases, vascular resection and reconstruction may be required [6]. In any case, the tumor’s size, location, degree of vascularization, proximity/adhesions to major vessels and surrounding tissues, and loco-regional invasion may guide the decision to perform each approach and may result in the conversion of laparoscopy to laparotomy [3,15]. Cytoreductive surgical resection, chemotherapy, radiotherapy, thermal ablation, and use of targeted radiolabeled carriers (e.g. ^131^I-MIBG for tumors that exhibit uptake on diagnostic scan) are viable treatment options for metastatic disease [4,11].

Histopathologic findings can usually confirm the diagnosis of a retroperitoneal paraganglioma, as the tumor microscopically shows the classic zellballen pattern, formed by well-developed, polygonal chromaffin cells surrounded by an intervening fibrovascular stroma and peripheral sustentacular cells [10]. Immunochemistry can demonstrate that the chief tumor cells are positive for neuroendocrine markers like chromogranin and synaptophysin, while sustentacular cells are positive for S100 protein stain, as observed in our case [13]. However, discriminating between benign and malignant lesions is challenging [10]. Absolute criteria to characterize a mass as malignant are extensive loco-regional invasion and presence of metastasis in organs where chromaffin tissue is normally absent [5]. Growth patterns, mitoses, and atypia of cells and nuclei are used to predict the clinical behavior of paragangliomas and are incorporated in scoring systems like the GAPP system [11]. In our case, the mass was of intermediate differentiation according to the GAPP system; however, the GAPP system’s utility in predicting future metastasis has not been proven yet [10]. Ki67 is a marker that has been widely used to predict malignancy, but the large numbers of false-positive and false-negative results at individual levels have been a major drawback [10].

The prognosis of retroperitoneal paraganglioma is good in the case of a completely resected benign lesion and poor in the case of a malignant one [2]. According to the literature, malignancy is encountered in 20-42% of cases and malignant tumors can metastasize via lymphatic and hematogeneous route, while there is also a high incidence of local recurrence [5]. Regional lymph nodes, liver, bones, and lungs are the most common sites of metastatic spread [5]. In any case, life-long follow-up is recommended in order to detect metastasis or recurrence on time [8,13].

## Conclusions

Non-functional, extra-adrenal, retroperitoneal paraganglioma poses a real diagnostic and surgical challenge. Growing evidence indicates that minimally invasive surgical procedures are effective and safe in the treatment of such lesions. A multidisciplinary medical approach and treatment planning are of paramount importance to achieve optimum outcomes for the patients, both oncologically and with regard to perioperative complications.

## References

[1] Heinze A, Nikomanis P, Petzold F, Rassweiler JJ, Goezen AS (2019). Undiagnosed paraganglioma; a challenge during laparoscopic retroperitoneal resection. Arch Ital Urol Androl.

[2] Ikram A, Rehman A (2020). Paraganglioma. https://www.ncbi.nlm.nih.gov/books/NBK549834/.

[3] Alemanno G, Bergamini C, Somigli R, Prosperi P, Bruscino A, Valeri A (2017). Abdominal paragangliomas: a quantitative prognostic score as predictive factor of the feasibility of the laparoscopic approach. Updates Surg.

[4] Gannan E, van Veenendaal P, Scarlett A, Ng M (2020). Retroperitoneal non-functioning paraganglioma: a difficult tumour to diagnose and treat. Int J Surg Case Rep.

[5] Wen J, Li HZ, Ji ZG, Mao QZ, Shi BB, Yan WG (2010). A decade of clinical experience with extra-adrenal paragangliomas of retroperitoneum: report of 67 cases and a literature review. Urol Ann.

[6] Bacalbasa N, Balescu I, Tanase A, Brezean I, Vilcu M, Brasoveanu V (2018). Successful resection of a non-functional paraganglioma with celiac trunk invasion followed by common hepatic artery reimplantation - a case report and literature review. In Vivo.

[7] Parmar K, Chandna A, Kumar S (2019). Retroperitoneal paraganglioma: a chameleon masquerading as an adrenal pheochromocytoma. Ann R Coll Surg Engl.

[8] Brahmbhatt P, Patel P, Saleem A, Narayan R, Young M (2013). Retroperitoneal paraganglioma presenting as a chest pain: a case report. Case Rep Oncol Med.

[9] Jawad Z, Fajardo-Puerta AB, Lefroy D, Todd J, Lim PB, Jiao LR (2017). Complete laparoscopic excision of a giant retroperitoneal paraganglioma. Ann R Coll Surg Engl.

[10] Falhammar H, Kjellman M, Calissendorff J (2018). Treatment and outcomes in pheochromocytomas and paragangliomas: a study of 110 cases from a single center. Endocrine.

[11] Neumann HPH, Young WF Jr, Eng C (2019). Pheochromocytoma and paraganglioma. N Engl J Med.

[12] Lin WC, Wang HY, Chang CW, Lin JL, Tsai CH (2020). Retroperitoneal paraganglioma manifesting as paralytic ileus: a case report. J Med Case Rep.

[13] Chattoraj AK, Rao UM, Sarkar N, Jakka S (2019). Non-functional retroperitoneal paraganglioma: a case report. J Family Med Prim Care.

[14] Zhang B, Chen Z, Hu X (2018). Application of three-dimensional visualization technology in laparoscopic surgery for pheochromocytoma/paraganglioma: a single-center experience. J Laparoendosc Adv Surg Tech A.

[15] Xu W, Li H, Ji Z, Yan W, Zhang Y, Zhang X, Li Q (2020). Retroperitoneal laparoscopic management of paraganglioma: a single institute experience. PLoS One.

